# Protein-Based Biomaterials for Bio-Optoelectronics: A Review of Bio-Integrated and Biodegradable Optodevices

**DOI:** 10.3390/mi17101118

**Published:** 2026-09-24

**Authors:** Anna Merlo, Fosca Conti

**Affiliations:** 1 Department of Pharmaceutical Sciences, University of Padova, Via Marzolo, 5, 35131 Padova, Italy; anna.merlo.5@studenti.unipd.it; 2 Department of Chemical Sciences, University of Padova, Via Marzolo 1, 35131 Padova, Italy

**Keywords:** biodevice, biomedical, bioimplants, biohybrid, sensing, imaging, neuromodulation, biotech, protein, biosensor

## Abstract

Growing development of biomaterials has stimulated the biomedical sector to seek innovative alternatives to conventional medical technologies. Bio-optoelectronic devices are emerging as promising tools, integrating biologically derived components, biodegradable end-of-life approaches, and highly biocompatible materials with optoelectronic systems. The devices combine optical and electronic properties with biological interfaces, enabling more effective interaction with tissues and cellular systems. Applied research is motivated by the need to improve the efficacy and biocompatibility of medical devices while reducing invasiveness and adverse effects. Wearable devices and implants based on flexible, biodegradable, or bioinspired materials allow better adaptation to biological tissues and real-time monitoring or modulation of physiological processes. Proteins are attracting increasing attention due to their biocompatibility, biodegradability, mechanical flexibility, and ability to respond to optical and electrical stimuli. Furthermore, the combination of proteins with synthetic materials and nanomaterials in biohybrid platforms offers new opportunities for advanced bio-optoelectronic systems. This review summarizes key developments in bio-optoelectronic devices, focusing on protein-based materials; their roles in sensing, imaging, and neuromodulation; and their use in wearable, implantable, and biotech systems. Crucial challenges, including long-term stability and integration with biological systems, are also discussed, together with future perspectives for diagnostic and therapeutic applications.

## 1. Introduction

Developing materials for biomedical applications has been the focus of continuous and advanced research. Indeed, the materials traditionally used often show undesired rigidity and limited biocompatibility, which can trigger inflammatory responses and foreign-body reactions. In the context of bioelectronic devices, softer materials, such as organic elastomeric polymers and hydrogels, have been explored as promising substrates due to their mechanical properties and potential to better match the requirements of biological tissues [1]. Indeed, the development of materials capable of combining suitable mechanical properties with biological compatibility and specific functional properties is of particular interest for the design of next-generation biomedical devices [2]. Bio-optoelectronic devices have been highlighted as a promising class of systems that integrate biological components with optoelectronic functionalities to enable the detection, modulation, and transduction of light within biological environments [3]. Biomedical applications include sensing, stimulation, imaging and therapeutic applications [4]. Bio-optoelectronic systems can be developed from a wide range of materials, including inorganic semiconductors, organic systems, nanomaterials, and, more recently, biomolecular platforms, each associated with different strengths and limitations [5]. In particular, inorganic materials can provide excellent optical and electronic performance, while organic materials offer greater flexibility and processability [6,7,8]. Nanomaterials have also been extensively investigated owing to their unique surface properties [9,10]. However, despite many advantages, the integration of these materials with biological systems may still be limited by issues such as mechanical mismatch, biocompatibility, biodegradability, and long-term stability. Among material platforms investigated for bio-optoelectronic applications, protein-based systems have gained interest thanks to their intrinsic biocompatibility, structural tunability, and ability to perform highly specific light-driven functions. Compared to conventional optoelectronic technologies, which are often characterized by mechanical mismatch with biological tissues, limited biodegradability, and long-term integration, protein-based materials offer an innovative approach for in vivo interfacing [11,12]. Furthermore, the possibility of engineering proteins’ structure and function may enable the development of materials with tailored properties for specific applications [13,14]. This review provides an overview of the current state of bio-optoelectronic devices, with a specific focus on protein-based platforms, highlighting their working principles, advantages, and current challenges as well as identifying future directions for their development and application in biomedical fields.

## 2. Bio-Optoelectronic Devices

Bio-optoelectronic devices integrate electronic, optical, and biological elements into a unified platform capable of sensing and processing biological information. These devices can be considered an evolution of conventional biosensors. In biosensors, a biological recognition element is coupled to a physical transducer that converts a biochemical signal into a measurable output parameter [15]. In bio-optical electronic devices, optical phenomena such as absorption and emission of radiation are read and processed by the electronic component of the device.

A generic device is characterized by three main components: an electronic module, an optical module, and a biological unit. The electronic module is made up of semiconductor materials, electrodes, and processing circuits; its function is to generate and amplify electrical signals by converting biological or optical signals into measurable electrical signals, ensuring the processing and reading of the result of a perturbation [16]. The optical module is represented by light sources, photoactive materials, and optical sensors. This part generates light radiation, transporting information from the biological system to the electronic system in the form of optical radiation. It is a non-invasive interface, because it allows the analysis of biological systems without the need for direct electrical contact or destructive sampling, thereby preserving the structural and functional integrity of the sample while still providing highly sensitive measurements [17]. Finally, the biological unit consists of biomolecules, cells, and materials of biological origin and allows for the selective recognition of analytes and processes. The unit reacts to optical or electronic stimuli, guaranteeing a selectivity and rapidity that cannot be achieved with purely synthetic materials. Generally, in bio-optoelectronic devices, the biological unit interacts with the environment, and this interaction results in a change in the optical or electronic properties of the unit. Moreover, changes in optical signals generated by cells or tissues can be detected in real time and used as feedback signals to control subsequent optical stimulation or manipulation. For example, online two-photon imaging of neuronal activity has been used to dynamically adapt optogenetic stimulation in vivo, establishing a closed-loop system for controlling neural circuit activity [18]. Similarly, chemical-selective fluorescence signals have been detected in living cells and used to control laser-induced intracellular molecular activity through a closed-loop optoelectronic feedback system [19]. The optical components convert the variations in light radiation to stimulate the biological system or to translate a result. Finally, the electronic components translate the signal into a readable or analyzable output [20]. The integration of these three parts ensures greater sensitivity, supports devices miniaturization, and allows real-time monitoring. Therefore, these devices are very attractive in the biomedical sector.

Figure 1 illustrates the interdependence between the three main parts of the device: (i) optical components, responsible for light generation and manipulation (LEDs, lasers, photodetectors) and signal transduction (photodetectors) [21,22,23]; (ii) electronic components, intended for data processing, signal amplification, and power management throughout integrated circuits and microcontrollers; and (iii) biological components, which represent the target or functional element of the system (cells, biomarkers, DNA) [24]. Systems involving only two of these three parts can also be identified. The biological–optical interface includes systems in which light is directly used to study or manipulate biological components, as demonstrated by all-optical closed-loop voltage control of neurons and muscle cells using fluorescent voltage indicators and optogenetic actuators [25]. The biological–electronic interface includes devices in which biological cells or tissues are directly interfaced with electrodes, such as microelectrode arrays, which are primarily used for extracellular measurements. In specialized configurations, the interface can also enable intracellular recording from neuronal cultures [26]. However, intracellular measurement remains a difficult or temporary process because of cell penetration. Conversely, the optical–electronic interface comprises systems in which optical signals are detected and processed by electronic components without a direct biological component, as demonstrated by optoelectronic arrays integrating photodiodes with resistive random-access memories for in-sensor computing [27]. The central overlapping area in Figure 1 highlights the cooperation of the three modules required for real-time monitoring and advanced diagnostics, where biological signals are converted into optical signals and subsequently electronically processed.

A critical component of bio-optoelectronic devices is the biophysical interface between the artificial system and the biological tissues. The interface must ensure efficient coupling between electronic and optical signals and biological processes. Therefore, depending on the device’s architecture, it may involve ionic transport, electrochemical interactions, and electromagnetic coupling. Specifically, in bioelectronics interfaces based on direct electrical coupling with cells and tissues, communication with cells and tissues occurs through the conversion between ionic signals of biological systems and electronic signals of artificial devices, requiring biocompatible and mechanically conformable materials [28,29]. In bio-optoelectronic systems, this electrical/ionic coupling can be combined with optical transduction, in which light is used to examine, stimulate, or modulate biological systems. For example, optoelectronic neural interfaces can integrate optical stimulation with electrical recording, thereby establishing both optical and electronic interfaces with biological tissue [30]. At the same time, optical interaction enables non-invasive analysis of biological systems, exploiting light absorption, emission, and scattering processes in tissues [31]. To ensure effective and reliable bio-optoelectronic interfaces, biomaterials should meet a combination of requirements related to their interaction with biological tissues and their functional performance. These include mechanical compatibility and conformability to facilitate intimate contact with tissues while minimizing mechanical mismatch [32]; physiological stability and biocompatibility to preserve material integrity and functionality under relevant physiological conditions while limiting adverse biological responses [28]; and suitable optical and electronic properties to enable efficient interaction with and transduction of biological signals [33]. In addition, processability and structural tunability are important for tailoring material properties to specific device architectures and biomedical applications. Finally, biodegradable and/or bioresorbable materials, when required, can facilitate transient applications and minimize the need for long-term device removal [34,35]. Together, these requirements provide a general framework for the design and selection of biomaterials for bio-optoelectronic applications.

## 3. Materials for Bio-Optoelectronics

The development of soft devices has been driven by the need to design tools and materials that are mechanically compatible with biological tissues, which typically exhibit a Young’s modulus ranging from 1 to 4 kPa for brain tissue to 1 to 4 MPa for skin. The softness reduces the invasiveness and adverse effects associated with traditional rigid materials [36]. Figure 2 shows the materials generally used in bio-optoelectronic devices; they can be divided into four main categories: inorganic materials, organic polymers, bio-based natural materials, and nanomaterials [5].

Inorganic materials such as silicon, metal oxides, and conventional semiconductors are known for their excellent electronic properties and high optoelectronic efficiency [6,7]. However, these materials are intrinsically poorly biocompatible, with the risk of inducing inflammatory responses when implanted into biological systems [1,5]. In order to overcome these limitations, miniaturization and material-engineering strategies have been developed, such as the use of bioresorbable silicon, capable of progressively dissolving in a physiological environment [37]. Miniaturization, achieved through reducing device dimensions to the micro and nanoscale, allows for better integration with biological systems, reducing invasiveness and enhancing spatial resolution in bio-optoelectronic applications [9].

Organic polymers offer high flexibility, softness, and the ability to adapt effectively to biological tissues; some examples include polymethyl methacrylate (PMMA), polyethylene (PE), and conjugated semiconductor polymers, such as PEDOT:PSS and polypyrrole (PPy) [28,38]. These materials can provide different functionalities depending on their chemical and electronic properties and their specific role within the device. In particular, PMMA and PE are mainly used as insulating, mechanically flexible, and optically transparent materials, whereas conjugated polymers such as PEDOT:PSS and PPy can provide efficient electrical charge transport and electrochemical activity [2,8,38]. PMMA, despite being an electrical insulator, can also be used as a dielectric layer in organic electronic devices, where it electrically isolates the active channel while enabling gate modulation [39]. In optoelectronic devices, PMMA can serve as a transparent matrix or passivation/interface layer rather than as the active light-emitting or light-detecting material. For instance, PMMA has been employed as a passivation layer in photodetectors, where it reduces interfacial defects, leakage, and noise currents, thereby improving the devices’ detectivity [40]. Similarly, PMMA can act as a host or encapsulation matrix for optically active materials, while the embedded active component is responsible for light emission [41]. In electronics, conjugated and conductive polymers such as PEDOT:PSS and PPy are therefore particularly relevant for efficient charge transport, electrical signal transmission and bioelectronic interfacing, while light emission and detection generally arise from conjugated semiconductor or hybrid active materials integrated with the polymer matrix or interface [2,8]. Finally, in bioelectronic devices, PEDOT:PSS and PPy can form an electrically active interface with cells and tissues owing to their conductivity, electrochemical activity, mechanical compliance and compatibility with soft biological interfaces [2,41]. These characteristics have led to their widespread use in wearable and implantable technologies.

Natural biomaterials, including collagen, chitosan, silk, and photosensitive proteins, play a crucial role due to their intrinsic biocompatibility, biodegradability, and ability to be recognized by biological systems [11,42,43,44,45]. In bio-optoelectronic systems, however, their role extends beyond serving as passive scaffolds for cell growth, as some nature-derived materials can also provide functional optical, electrical, or electrochemical properties at the interface with biological systems [42]. In bioelectronic applications, these materials are used both as scaffolds for cell growth and as active components, either intrinsically or in combination with conductive and optically active materials [42]. For example, silk fibroin can provide mechanically compliant and biocompatible interfaces while also serving as a functional material for bioimaging and biosensing applications [45]. Similarly, photosensitive proteins such as bacteriorhodopsin can directly participate in the conversion of optical stimuli into electrical signals, enabling light-responsive bio-photoelectric sensing [46]. Thus, rather than acting solely as structural supports, selected natural biomaterials can contribute directly to the coupling between optical, electrical, and biological signals, while others primarily provide the biocompatible structural environment required for the integration of active components.

Nanomaterials have also attracted interest in regard to biomedical devices; they are defined by their nanometer dimensions, high surface area, and distinctive size- and structure-dependent electronic, optical, and surface properties. Among them, two-dimensional (2D) nanomaterials, including graphene; transition metal dichalcogenides (TMDs) such as MoS_2_ and WS_2_; black phosphorus; MXenes; and hexagonal boron nitride (h-BN), exhibit a broad range of electronic and optical properties. Graphene provides high electrical conductivity, a large surface area, mechanical flexibility, and sensitivity to surface interactions, whereas semiconducting TMDs such as MoS_2_ and WS_2_ offer thickness-dependent band structures and strong light–matter interactions. Black phosphorus exhibits a tunable bandgap and anisotropic optical properties, while MXenes combine high electrical conductivity with chemically tunable surfaces [10,47,48]. Quantum dots and nanowires represent additional nanomaterial classes with distinctive optoelectronic and electrical properties [9,10]. The physical–chemical characteristics and high surface-to-volume ratios of these materials make them particularly suitable for the creation of highly sensitive bioelectronic interfaces and biosensors, which are capable of detecting biological signals with high precision [49].

An important parameter for evaluating the suitability of materials for bio-optoelectronic applications is the Young’s modulus, which describes their mechanical stiffness. As shown in Table 1, conventional inorganic materials exhibit significantly higher Young’s moduli than biological tissues, resulting in a pronounced mechanical mismatch. In contrast, organic polymers and biomaterials display values that are closer to those of native tissues, promoting improved mechanical compliance and biocompatibility [1,50,51]. However, mechanical compliance represents only one of the requirements for bio-optoelectronic applications. In Table 1, six classes of materials are listed together with a representative sample. The different material classes provide complementary advantages in terms of electrical conductivity, optical functionality, biocompatibility, biodegradability, processability, and the ability to interface with biological tissues. Here, mechanical compliance refers to the ability of a material to accommodate deformation and bending and conform to biological tissues, while optical functionality refers to the ability to support optical or optoelectronic functions relevant to bioelectronic applications, i.e., interaction with and processing of light. Qualitative assessments of material parameters in the context of bioelectronics are summarized with different symbols in Table 1: √ indicates an intrinsic characteristic or a generally promising response for bioelectronic interfacing; ~ indicates a characteristic that depends strongly on material composition, formulation, processing, or device architecture; and × indicates a generally disadvantageous characteristic. For example, silicon is a semiconductor with relatively low electrical conductivity but offers excellent electrical and optical performance after doping; however, silicon exhibits a substantial mechanical mismatch with soft tissues. However, organic polymers provide greater flexibility but are generally electrically insulating unless specifically engineered to be conductive. Natural and protein-based biomaterials offer excellent biocompatibility and biodegradability but typically require integration with electrically or optically active components to provide efficient signal transduction. Nanomaterials, including 2D materials such as graphene and MoS_2_, can provide high electrical and optoelectronic performance together with mechanical flexibility and large surface areas, although their long-term biocompatibility and integration with biological systems remain material- and application-dependent [10,49].

Although each material class offers several advantages, no one class individually satisfies all the specific requirements for bio-optoelectronic applications, making hybrid solutions an attractive approach. Rather than indicating that all materials require extensive development across multiple parameters, the complementary nature of these properties suggests that hybrid systems can compensate for specific limitations while preserving the intrinsic advantages of each component. Hybrid systems are designed to combine complementary properties such as electrical conductivity, mechanical flexibility, biocompatibility, and optical response [2]. Each class contributes specifically to the overall performance of the device: inorganic materials and nanomaterials ensure high optoelectronic performance, organic polymers provide flexibility and processability, and natural biomaterials facilitate interaction with biological systems. In particular, biomaterials are not limited to a structural role but can be designed to actively interact with biological components, including biofunctional molecules [42]. Proteins in this scenario play a key role due to their high molecular specificity and ability to respond to external stimulations. Therefore, proteins represent a class of functional biomaterials capable of directly participating in transduction processes in bioelectronic devices.

## 4. Roles of Proteins in Bio-Optoelectronic Devices

Proteins represent one of the most versatile classes of biomolecules thanks to the high chemical diversity of their constituent amino acids and the complexity of their three-dimensional structures [53,54]. Moreover, the presence of multiple functional groups, including aromatic residues and redox centers, gives proteins heterogeneous and tunable electronic properties. Aromatic amino acids such as tryptophan, tyrosine, and phenylalanine participate in UV absorption processes and electronic energy transfer mechanisms [55]. Numerous studies have shown that specific proteins can exhibit optoelectronic properties relevant for applications in bio-optoelectronic devices [11,12]. However, the suitability of a protein-based material for bio-optoelectronic applications depends on the specific combination of its structural, mechanical, physiological, and optoelectronic properties [56]. Key parameters to consider include mechanical compliance, physiological stability, optical response, electronic functionality, biological activity, and processability, with their relative importance depending on the intended application [56]. For example, proteins with intrinsic light-responsive properties are particularly suitable for optical sensing and signal transduction, whereas proteins with favorable interfacial or structural properties can be exploited as functional layers, substrates, or interfaces in biohybrid devices [57,58]. Therefore, the selection of a specific protein should be guided by the requirements of the target application and by the balance between its functional properties and its stability under the intended operating conditions [56].

Photosensitive proteins, such as microbial rhodopsins, and fluorescent proteins are able to transduce optical stimuli through different molecular mechanisms. Fluorescent proteins primarily absorb excitation light and re-emit it at a longer wavelength, providing an optical-to-optical transduction mechanism widely exploited for fluorescence-based imaging and sensing [59]. In contrast, microbial rhodopsins can convert absorbed light into electrochemical signals through their photochemical cycles. For example, bacteriorhodopsin (BR) undergoes light-induced retinal isomerization and conformational changes coupled with proton translocation, generating an electrogenic response that can be exploited for photoelectric signal transduction [60,61,62]. Furthermore, proteins can exhibit photoconductivity reactions, such as an increase in electrical conductivity under illumination, as observed in protein films containing active chromophores [61]. Importantly, conformational changes can have different roles depending on the protein system: in microbial rhodopsins, they are intrinsic to the photocycle and contribute to proton pumping [62], whereas in fluorescent-protein-based biosensors, they can be coupled with changes in fluorescence. For example, analyte-induced molecular rearrangements can modulate FRET in fluorescent-protein-based biosensors [63], while genetically encoded voltage indicators use voltage-dependent changes associated with microbial rhodopsin domains to optically report membrane potential [64]. The potential biocompatibility of appropriately selected and engineered proteins, together with their ability to work in physiological conditions, make proteins particularly promising for biomedical applications. Indeed, coupling optical, electrical, and biological signals at the biointerface makes proteins capable competitors with respect to traditional semiconductors [65].

Representative examples illustrate how these protein-based systems can provide measurable optoelectronic responses. BR immobilized on thin films on electrodes has shown photoconductive behavior and highly reproducible photoelectric responses. In particular, optimized bacteriorhodopsin films generate photocurrent peaks of up to 5.38 nA over an illuminated area of 3.14 mm^2^ (out of a total film area of 78.54 mm^2^). Concerning the response over time, this protein showed no significant decrease in peak photocurrent after three weeks, indicating a stable and reproducible photoelectric response [61]. Furthermore, fluorescent proteins, particularly green fluorescent protein (GFP), are present in many small water organisms. Even if their structures and mechanisms of chromophore formation are quite complex, GFPs are widely used as biological markers and biosensors in cell and molecular biology. GFP-functionalized individual semiconducting carbon nanotubes have been shown to also exhibit charge transport and semiconductor behavior. For individual field-effect transistors based on semiconducting single-walled carbon nanotubes (SWCNT-FETs) functionalized with variants of superfolder GFP, sfGFP^132azF^, and sfGFP^204azF^, a decrease in conductivity was observed after sfGFP attachment. Photo-responsivities up to 7 × 10^3^ A W^−1^ and current modulation reaching 15-fold under visible-light excitation were reported [66].

Protein engineering allows for the targeted modulation of the structural and electronic properties of proteins. Techniques such as site-directed mutagenesis allow for the modification of the amino acid sequence in order to obtain specific functionalities [67]. Also, targeted mutations can modify the absorption spectrum, determining shifts towards red or blue, as well as influence fluorescence quantum efficiency, thermal and chemical stability, and the electron transfer rate. In the case of microbial rhodopsins, modifications of the chromophore site have been made in order to generate proteins with customized spectral responses, which are also widely used in optogenetics [68]. Fluorescent proteins have also been engineered to cover a broad emission spectrum and improve light output [69]. Protein engineering has also enabled the development of light-switchable proteins for spatiotemporal control of specific biological processes, as demonstrated for engineered RNA-binding proteins [70]. An emerging field involves the de novo design of proteins with specific electronic properties, in which artificial sequences are developed to facilitate charge transport or interaction with electrodes [13,14]. Overall, proteins can be exploited in bio-optoelectronics through their intrinsic optoelectronic properties, through engineering approaches aimed at tailoring their structures and functionality, or through their integration with inorganic and nanostructured materials to form biohybrid interfaces. These strategies enable the coupling of biological, optical, and electronic functions and can be adapted to the requirements of specific applications. A schematic overview of these approaches is presented in Figure 3.

Research is increasingly focused on the development of biohybrid systems, which combine proteins with inorganic or synthetic organic materials to create devices with improved performance and novel functionalities. Proteins can act as active elements, such as photosensors, or as interfaces between biological and electronic components [2]. Successful applications include protein–electrode devices, in which redox-active proteins are immobilized on conductive surfaces such as gold, graphene, or semiconductor oxides to create biosensors [71,72,73,74]. Other approaches include nanostructured protein films, used as active layers in optoelectronic devices, as well as hybrid protein–nanomaterial systems, for example, with metal nanoparticles or quantum dots, which exploit plasmonics or energy transfer mechanisms [75,76]. The different strategies aim to exploit the specificity of proteins while retaining the stability and processability of synthetic materials.

Regarding advanced materials, the integration of proteins with graphene and other 2D materials has shown significant improvements in electronic coupling and device stability. Specifically, Hassan et al. [77] highlighted the strong potential of graphene-based components and transition metal dichalcogenides (TMDCs), such as MoS_2_, for highly sensitive optoelectronic and biosensing applications. In these hybrid systems, proteins can provide biological recognition or light-responsive functions, while the 2D material acts as the electrical transducer: biological binding or light-induced changes in the protein can modulate charge or energy transfer at the protein/2D-material interface, resulting in a measurable electrical or optical signal. This coupling enables 2D materials to directly connect biological or optical events with electronic readouts rather than serve only as conductive substrates [77].

## 5. Applications of Protein-Based Bio-Optoelectronics

Protein-based bioelectronic devices have been investigated in different fields, including biosensing [78], imaging [79], and neuromodulation [80,81]. Biosensors exploit biomolecules such as enzymes, receptors, and fluorescent proteins as highly selective recognition elements. Proteins can specifically (i) bind analytes, e.g., neurotransmitters or glucose; (ii) induce changes in optical properties, such as fluorescence; or (iii) generate electronic signals, especially when integrated with semiconductor materials [71]. Therefore, protein-based bioelectronic devices are widely used in medical diagnostics [82] but also for environmental monitoring and real-time analysis of biological systems [83].

Fluorescent biosensors can be applied in the study of neurotransmission because they enable measurement with high temporal and spatial resolution, thus monitoring neuronal activity in vivo [84,85]. Another application involves photoelectrochemical immunosensors based on recognition proteins combined with nanostructured semiconductors for tumor biomarker detection. Wang et al. [86] created a photoelectrochemical biosensor using TiO_2_ nanosheets and carboxylated graphitic carbon nitride functionalized with anti-carcinoembryonic antigen antibodies. Under light irradiation, the system generated a photocurrent that changed according to the concentration of carcinoembryonic antigen. The semiconductor structure improved charge separation and photoelectric conversion, allowing detection in the picogram-per-milliliter range. These photoelectrochemical biosensor systems can operate as self-powered platforms, where light irradiation directly generates an electrical response in the presence of the target analyte. This feature reduces external power requirements and supports highly sensitive diagnostic applications.

In the field of imaging, advanced improvements have been made thanks to the use of proteins. In particular, fluorescent proteins and genetically encoded sensors have improved biological imaging techniques because of their high specificity and the possibility of non-invasive, real-time imaging [84]. Some of these sensors rely on neurotransmitter-binding proteins coupled with optical-fiber-based platforms. Binding events induce conformational changes that modify fluorescence emission, allowing the detection of neurotransmitter dynamics in vivo [84,87]. Other techniques such as optical microscopy and miniaturized imaging allow for the observation of biological processes with great precision [87]. A recent example concerns the development of mitochondrial fluorescent probes capable of simultaneously detecting physiological changes within living cells [88]. Indeed, optical transduction converts intracellular biochemical changes into measurable fluorescence variations, supporting real-time monitoring of cellular conditions. Li et al. [88] developed a mitochondrion-targeted fluorescent probe that monitored two different parameters through separate fluorescence signals in vivo. Variations in viscosity and levels of reactive nitrogen species, such as peroxynitrite, are correlated with oxidative stress and mitochondrial dysfunction, so simultaneous detection may provide useful information on cellular conditions.

Regarding neuromodulation, photosensitive proteins offer extremely powerful tools for controlling neuronal activity through light stimulation, allowing the selective activation or inhibition of specific neuronal populations. This approach finds application both in basic research and in the development of experimental therapeutic strategies for neurological diseases [68,89,90]. Photosensitive proteins can be expressed in neurons, allowing extremely precise regulation of their activity. Among the photosensitive proteins used in optogenetics, channelrhodopsins are microbial rhodopsins that function as light-gated ion channels, enabling direct modulation of membrane potential through light-induced ion flux. Microbial rhodopsins should be distinguished from vertebrate rhodopsins, which are G-protein-coupled receptors (GPCRs): upon light-induced retinal isomerization, these receptors activate intracellular G-protein signaling cascades instead of directly conducting ions across the membrane [91]. As discussed above, proteins such as rhodopsins, upon light stimulation, can directly modulate ion flux across cell membranes, converting optical inputs into cellular electrical activity and therefore enabling precise control of neuronal signaling. Recent studies have also led to the development of engineered variants with greater light sensitivity and faster kinetics, further improving the efficacy of stimulation [92]. More recently, organic-semiconductor-based optoelectronic platforms, including flexible OLEDs and implantable OLED-on-CMOS probes, have been investigated for optogenetic neuromodulation. These systems allow integrated light delivery to neurons expressing photosensitive opsins and can enable high spatial and temporal control of neural stimulation [93]. Biohybrid artificial retinas are also being explored for vision restoration. In these systems, photoactive proteins are combined with electrode arrays to replace damaged photoreceptors and convert light into neural signals. This approach may support the recovery of visual function in retinal disorders [10].

Table 2 summarizes representative protein-based bio-optoelectronic devices and highlights the relationship between protein type, electronic interface, and optoelectronic function across biosensing, imaging, and neuromodulation applications.

## 6. Protein-Based Bio-Optoelectronics for Biomedical Devices

Protein-based bio-optoelectronic devices can be classified into three main families, based on the type of interface with the biological system and on the function they perform: wearables, implantable devices, and biotech systems. Moreover, the physical approaches can be classified into superficial, invasive, and biomimetic approaches [2,89,94].

Wearable bioelectronic and bio-optoelectronic devices are designed to operate at or near the body surface and can benefit from materials with high flexibility, conformability, and biocompatibility. Protein-based materials such as silk fibroin can be advantageous for wearable and implantable platforms because of their tunable mechanical, optical, and processing properties [95]. The mechanical and optical properties of silk fibroin are strongly influenced by its molecular conformation, aggregation state, and processing conditions. In particular, the conformational and assembly state of silk can affect the distribution and accessibility of reactive groups, thereby influencing the efficiency of subsequent chemical functionalization and the resulting material functionality [96]. Moreover, silk fibroin can be processed into nanostructured hydrogels with tunable transparency and mechanical properties, highlighting the role of processing conditions in tailoring its performance for bio-optoelectronic applications [97]. These features should be regarded as enabling characteristics rather than universal requirements for bio-optoelectronic devices since conventional wearable platforms can perform optical physiological sensing without relying on protein-based materials. The structure of silk-based materials can also influence the biological interface: for example, anisotropic silk nanofiber hydrogels have been shown to provide physical cues capable of regulating cell morphology and behavior [98]. Silk can also serve as a transparent and mechanically compliant optical interface, as demonstrated by silk-based opto-electro neural probes [95]. Silk fibroin has also been investigated in regard to wearable bioelectronic sensors for monitoring body movements and physiological parameters such as joint flexion, pressure, and muscle activity as well as in regard to electrochemical sensors for the detection of biomarkers in sweat [2,99]. These applications primarily exploit the mechanical, electrical, and chemical properties of the protein rather than an optical transduction mechanism. However, although silk fibroin has attracted considerable attention because of its high degree of biocompatibility and the lack of immune response when implanted within the body, maintaining long-term stability under physiological conditions remains a challenge [100]. The ability to produce a variety of forms and formats of scaffold types (sponges, hydrogels, coatings, films, electro-spun fibers, micro/nanospheres, etc.) offers a number of advantages over other biopolymer systems like collagen, chitosan, and alginate, for which there is less variety in processing choices. Gelatin has also been investigated as a flexible, transparent, and biocompatible material for implantable bioelectronics. Chen et al. [101] developed a gelatin-based conductive film whose high conformability enabled monitoring of tissue deformation, heartbeat, and respiratory signals. This application primarily relies on electrical and mechanical transduction rather than optical sensing. However, the use of hybrid systems seems to be the best strategy. Biohybrid devices based on nanoparticle-functionalized silk exhibit biomimetic catalytic activity and improved sensory performance, demonstrating the active role of proteins in signal transduction. Meng et al. described a wearable electrochemical sensor based on modified silk fibroin with gold nanoparticles (AuNPs) embedded in carbonized silk fibroin. The AuNPs provided peroxidase-like activity, improving the sensor’s sensitivity for H_2_O_2_ and glucose [76].

Implantable devices establish direct interactions with internal tissues and require key properties such as biocompatibility, in vivo stability, and controlled biodegradability. Optoelectronic neural probes made of silk fibroin are a significant example because they combine optogenetic stimulation and in vivo electrophysiological recording [95]. For instance, Zhou et al. [95] invented a so-called silk optrode consisting of a silk optical fiber integrated with flexible microelectrodes. The silk optical fiber provided a flexible and biocompatible optical interface for optogenetic stimulation, while the integrated microelectrodes enabled in vivo electrophysiological recording. Additionally, implantable bioelectronic devices based on modified silk have demonstrated the possibility of achieving long-term stable recordings thanks to structural engineering and controlled crystallization strategies. These approaches overcome the limitations of rapid degradation typical of protein biomaterials in physiological environments. Yang et al. [102] developed a silk-fibroin-based bioelectronic interface with controlled molecular orientation and crystallization. The silk structure was modified through a pre-stretching process that increased β-sheet formation. This strategy improved stability in aqueous environments and reduced structural degradation, allowing for a more stable in vivo recording.

Biotech systems are distinguished by the direct use of proteins as active elements in bioelectronic transduction, exploiting their electronic, optical, and molecular properties to mimic natural biological functions. The biodegradability of protein-based optoelectronic devices is also relevant and can be tuned through bioengineering and material-processing strategies. Target features can enable the creation of transient devices that degrade in the body after performing their function. This feature avoids the need for surgical removal and is particularly relevant for temporary applications. Hydrogel platforms and multilayer structures can be used to regulate device lifetime and degradation rate. By combining natural biopolymers with conductive materials, these devices maintain functionality during operation and gradually disappear after use. Applications include transient sensors and temporary implantable devices [103,104]. Fibroin-based protein microstructures have been exploited and developed for applications such as optical biosensing, wound monitoring, and controlled drug release [57,105]. In these systems, proteins may play either structural or functional roles, depending on the device architecture. In particular, silk fibroin can serve as a structural matrix or optical interface, while its optical properties or associated functional molecules can contribute directly to sensing and signal transduction. For example, Dong et al. [57] reviewed silk-fibroin-based optical microstructures for biomedical applications. The system used silk as both a structural platform and functional optical material, showing potential for both monitoring and therapeutic applications. Primary research has demonstrated that functionalized silk fibroin films can act as diffractive optical elements for optical sensing [104], while silk fibroin coatings on optical fibers can encapsulate fluorescent sensing molecules for real-time in vivo pH monitoring [106]. More recently, silk-fibroin-functionalized quantum-dot systems have been employed in smartphone-assisted fluorescent biosensing, further demonstrating the integration of silk-based materials with optical transduction mechanisms [105]. These examples illustrate how silk fibroin can play both structural and functional roles within bio-optoelectronic platforms.

## 7. Advantages, Limitations, Challenges, and Future Perspectives Regarding Protein-Based Bio-Optoelectronics

Protein-based bio-optoelectronic devices exhibit properties that make them particularly attractive compared to traditional electronic systems, including biocompatibility, biodegradability, and flexibility. The biological nature of proteins promotes more effective interaction with tissues, helping to reduce immune responses and improving integration into implantable systems [12,45,65]. Another point of strength is the possibility of creating biodegradable devices, which are designed to degrade in a controlled manner at the end of their life cycles, a feature particularly useful for temporary medical applications [103]. The mechanical properties of proteins, such as elasticity and deformability, combined with their processability into different configurations (films, hydrogels, fibers), also enable the creation of conformable devices that can adapt to complex biological surfaces. Proteins can be modified at the chemical or structural level, allowing the introduction of specific functionalities for sensing, actuation, and optoelectronic transduction applications [67,68]. The properties of proteins allow the development of multifunctional systems in which structural and functional roles are integrated, with potential applications that range from physiological monitoring to controlled drug release [57]. Sustainability is also a key advantage, as the use of naturally occurring materials contributes to reducing environmental impacts compared to conventional electronic technologies [103]. However, some critical issues still limit the application of these devices. Firstly, stability in a physiological environment remains a significant challenge, as proteins can undergo degradation processes or structural changes that compromise their performance over time [103]. Indeed, proteins are highly sensitive to temperature, humidity, and pH variations. Possible strategies involve protein engineering approaches and the development of protective matrices or encapsulation systems able to preserve protein conformation and functionality under operating conditions. Protein engineering can also be used to improve the stability of proteins under operating conditions. Changes in protein sequence and structure can help preserve protein functionality during device operation. Protective matrices and encapsulation can also be evaluated to reduce protein degradation. Long-term stability should be assessed through accelerated aging and real-time tests under physiological conditions [107]. Therefore, the optimization of protein design and green synthesis methods are becoming increasingly important to improve structural robustness without compromising functionality [108]. Moreover, the intrinsic variability of protein materials, linked to their biological origin and production methods, can affect device reproducibility and complicate large-scale standardization [109]. This variability can be reduced by controlling both the protein material and the fabrication process. Parameters such as protein purity, molecular weight, concentration, degree of functionalization, and water content can be monitored for each batch. Standardized protocols and quality-control procedures can then be used to improve reproducibility between batches. Recombinant production and protein engineering can also provide good control over protein sequences and composition, overcoming the need to use proteins obtained directly from biological sources [110,111]. Regarding performance, protein-based materials still present limitations compared to traditional semiconductors, particularly with respect to the electrical conduction and operational stability [103]. These issues also affect production scalability, long-term durability, and clinical validation, which are fundamental for a successful transition to industrial and biomedical applications [112]. For commercialization, fabrication methods should be adapted to larger production volumes while maintaining the properties of the protein material. Automated and scalable processes could reduce operator-dependent variability and improve production consistency. Manufacturing conditions should be optimized together with quality-control procedures and parameters for the final stage of device development. Sterilization, storage conditions, packaging, and shelf-life should be considered as well, especially for devices intended for biomedical applications. Other challenges include integration with micro- and nanofabrication technologies, which require conditions compatible with protein stability and therefore less aggressive processes than those used for inorganic materials [113]. Overall, the combination of protein engineering, standardized production, quality control, and scalable fabrication will be important for transitioning protein-based bio-optoelectronic devices from laboratory prototypes into practical and commercially viable applications [114].

## 8. Conclusions

Bio-optoelectronic devices show increasing potential for biomedical applications. Among the different materials investigated so far, proteins have gained particular attention because of their biocompatibility, biodegradability, and ability to respond to optical and electrical stimuli. Proteins can play different roles within bio-optoelectronic devices, acting not only as structural materials but also as active components involved in sensing, signal transduction, imaging, and neuromodulation. Proteins’ versatility allows the development of innovative biomedical technologies. Despite the advantages, several limitations still need to be addressed. Challenges related to long-term stability, reproducibility, large-scale production, and integration with existing technologies remain important obstacles to the wider implementation of protein-based devices. To address these challenges, future research should focus on improving protein stability through protein engineering, protective matrices, and encapsulation strategies. Moreover, standardized material characterization and controlled production methods could improve reproducibility. Accelerated aging and long-term in vivo studies will also be important to assess device stability and functionality under physiological conditions. Therefore, particular focus should be placed on the development of biohybrid platforms. Since each class of materials presents specific advantages and limitations, combining proteins with other materials represents an effective strategy to exploit the strengths of different components while compensating for their individual drawbacks. In particular, combining proteins with conductive, optical, or nanostructured materials could improve electrical performance, operational stability, and signal transduction while retaining the biological functionality of the protein component. Scalable and automated fabrication approaches should also be studied to facilitate reproducible production and integration with existing micro- and nanofabrication technologies. Finally, as a result, the design of more efficient and stable hybrid architectures represents one of the most promising directions for the advancement of bio-optoelectronic technologies, particularly for the development of biomedical technologies for diagnostic, monitoring, and therapeutic applications. In parallel, systematic evaluation of sterilization, storage, shelf-life, and long-term safety will be necessary to support the transition from laboratory prototypes into clinically relevant devices.

## Figures and Tables

**Figure 1:**
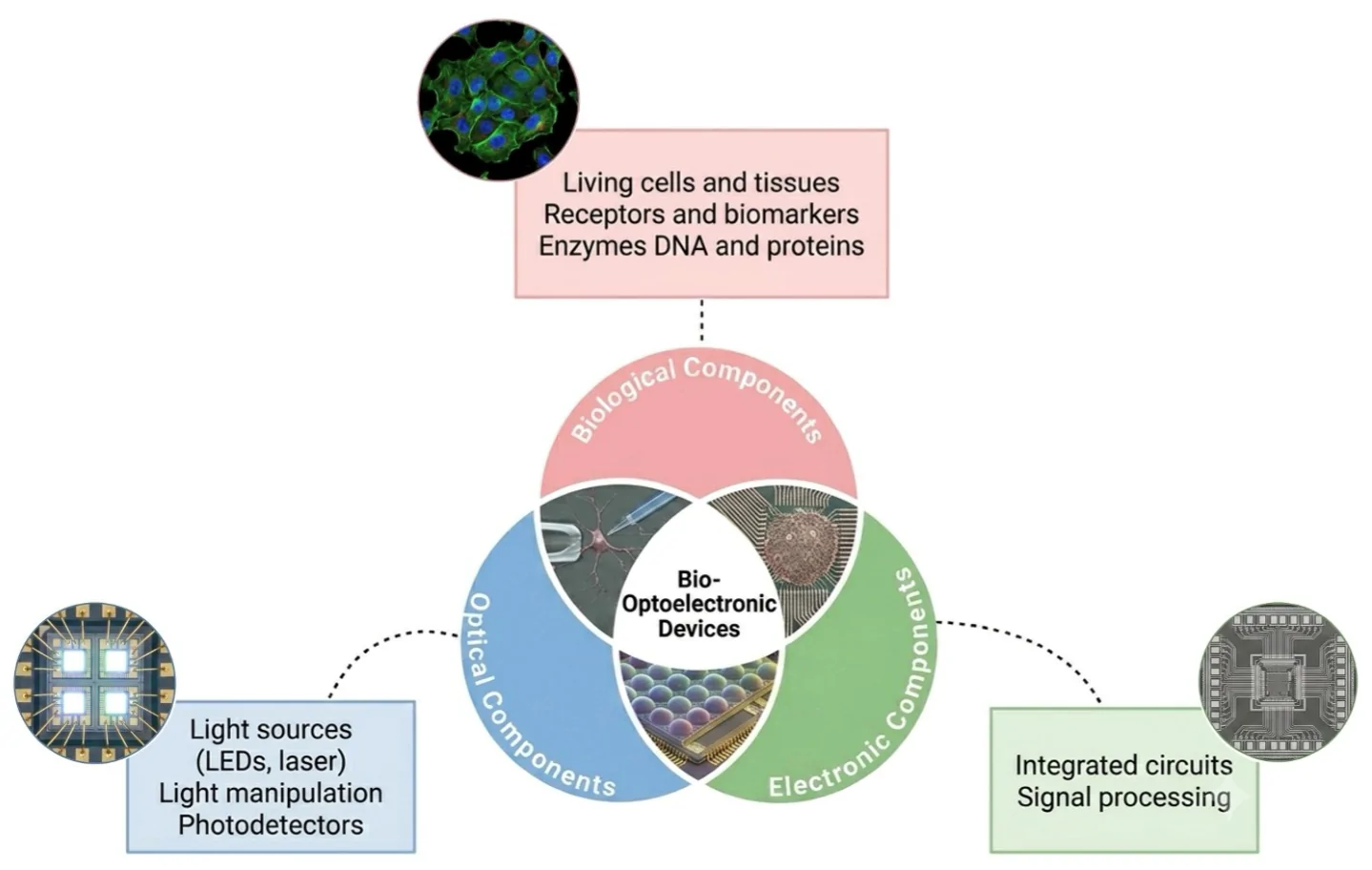
Schematic of the three functional domains of a bio-optoelectronic device: integration be-tween optical (sensing/transmission), electronic (processing) and biological (target/interface) units [21,22,23]. The images representing the biological, optical, and electronic components were generated using artificial intelligence (Gemini, version 3.6) for illustrative purposes.

**Figure 2:**
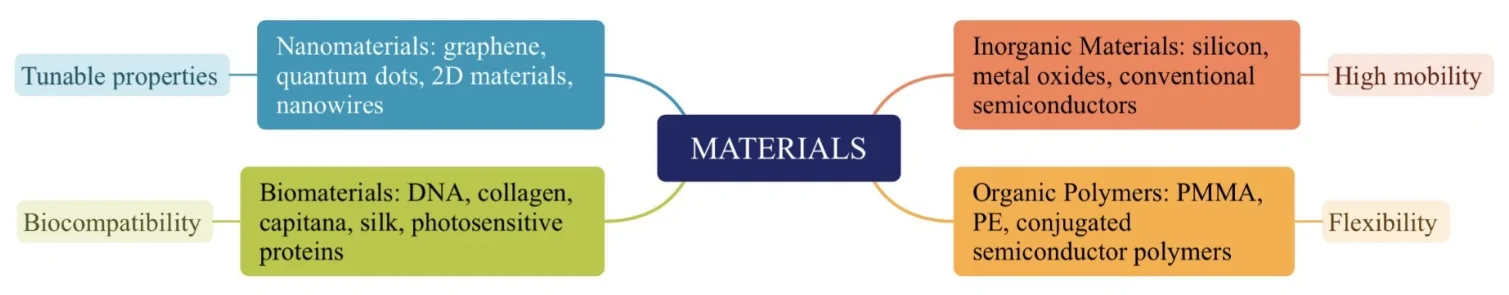
Classification of materials for bio-optoelectronic devices.

**Figure 3:**
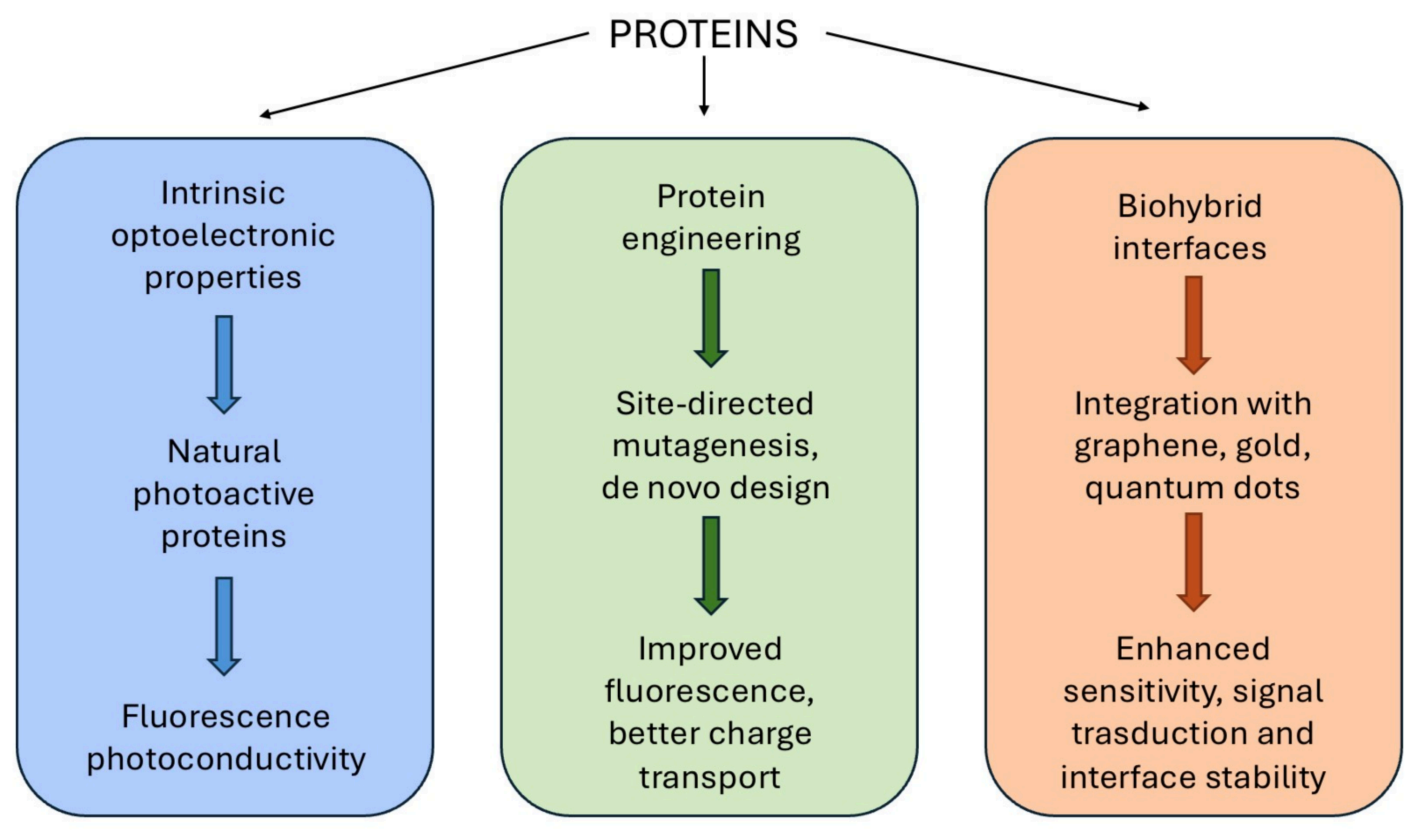
Overview of protein-based strategies in bio-optoelectronics.

**Table 1:** Young’s modulus and characteristics of some material classes useful in bioelectronics. ✓ denotes a promising/intrinsic characteristic; ~ denotes a characteristic that is material-composition-, formulation-, processing- or device-architecture-dependent; × denotes a generally disadvantageous characteristic.

Class of Materials	Representative Material and Corresponding Characteristics						
	Sample	Young’s Modulus Values of Untreated Samples	Mechanical Compliance	Electrical Conductivity	Optical Functionality	Biocompatibility	Reference
Biological tissues	Human brain tissue and human skin	1–4 kPa and 1–4 MPa	✓	~	✓	✓	[1]
Inorganic materials	Silicon	130–190 GPa	×	×	✓	~	[1]
Organic polymers	Polyethylene (PE)	0.1–1.2 GPa	✓	×	✓	✓	[50]
Natural biomaterials	Collagen Hydrogel	0.001–0.2 MPa	✓	×	~	✓	[51]
Protein-based material	Silk fibroin hydrogel	0.01–7 MPa	✓	~	✓	✓	[51]
Nanomaterials/2D materials	Graphene	ca. 1 TPa	✓	✓	✓	~	[52]

**Table 2:** Overview of protein-based bio-optoelectronic device architectures.

Device	Protein	Electronic Interface	Optoelectronic Function
Photoelectrochemical biosensor	Recognition proteins	Nanostructured semiconductors	Self-Powered: Generates an electrical signal from light in the presence of specific analyte
Fluorescent optical probe	Fluorescent proteins	Optical microscopy, imaging	Optic Transduction: Converts oxidative stress into fluorescence variations
Optogenetic neuromodulation implant	Photosensitive opsins	Optrodes, micro-LEDs, optical fibers/light guides	Light-induced modulation of neuronal activity
Bio-hybrid artificial retina	Photoactive proteins	Electrode arrays, neural circuits	Biointerface: Replaces damaged photoreceptors by converting light into neural signals
Optochemical sensor	Neurotransmitter-binding proteins	Optical Fiber system	Real-Time Monitoring: Translates chemical bonding into high-resolution light signals

## Data Availability

The original contributions presented in this study are included in the article/references. Further inquiries can be directed to the corresponding author.
